# Last Glacial Maximum led to community-wide population expansion in a montane songbird radiation in highland Papua New Guinea

**DOI:** 10.1186/s12862-020-01646-z

**Published:** 2020-07-11

**Authors:** Kritika M. Garg, Balaji Chattopadhyay, Bonny Koane, Katerina Sam, Frank E. Rheindt

**Affiliations:** 1 Department of Biological Science, National University of Singapore, 14 Science Drive 4, Singapore, 117543 Singapore; 2 The New Guinea Binatang Research Centre, Madang, Papua New Guinea; 3 Biology Centre CAS, Institute of Entomology, Branisovska 31, Ceske Budejovice, Czech Republic; 4 University of South Bohemia, Faculty of Science, Branisovska 1760, Ceske Budejovice, Czech Republic

**Keywords:** Quaternary glaciations, Scrubwrens, *Sericornis*, Demographic history, Genetic expansion

## Abstract

**Background:**

Quaternary climate fluctuations are an engine of biotic diversification. Global cooling cycles, such as the Last Glacial Maximum (LGM), are known to have fragmented the ranges of higher-latitude fauna and flora into smaller refugia, dramatically reducing species ranges. However, relatively less is known about the effects of cooling cycles on tropical biota.

**Results:**

We analyzed thousands of genome-wide DNA markers across an assemblage of three closely related understorey-inhabiting scrubwrens (*Sericornis* and *Aethomyias*; Aves) from montane forest along an elevational gradient on Mt. Wilhelm, the highest mountain of Papua New Guinea. Despite species-specific differences in elevational preference, we found limited differentiation within each scrubwren species, but detected a strong genomic signature of simultaneous population expansions at 27-29 ka, coinciding with the onset of the LGM.

**Conclusion:**

The remarkable synchronous timing of population expansions of all three species demonstrates the importance of global cooling cycles in expanding highland habitat. Global cooling cycles have likely had strongly different impacts on tropical montane areas versus boreal and temperate latitudes, leading to population expansions in the former and serious fragmentation in the latter.

## Background

Quaternary glacial cycles have shaped present species distributions and patterns of population differentiation across the globe [1, 2]. The effects of Quaternary glaciations differ widely across regions [2, 3]. For example, in the northern hemisphere, glacial ice sheets have covered substantial areas of suitable habitat, forcing many species into small refugial pockets [2, 4, 5]. In contrast, in many shallow coastal parts of the world, especially across Australasia, genetic connectivity among isolated populations may increase during periods of global cooling, as the global sea level drops by up to 120 m, allowing for the formation of land bridges and connectivity [1, 6–8]. However, research on the evolutionary impact of Quaternary glaciations on species-rich tropical mountains is still in its infancy.

Quaternary periods of global cooling are known to have forced many species of montane flora and fauna to periodically descend from mountains to lower elevations [2, 9, 10]. As a result, isolated montane populations may routinely undergo demographic expansion due to an increase in suitable cold-adapted habitat during ice ages [2, 11, 12], sometimes even allowing for contact and genetic exchange between neighboring mountains [10, 13–15]. However, what is lacking so far is a rigorous population-genomic test of this hypothesis using multiple members of a radiation co-inhabiting the same mountains, especially in the Old World.

In this study, we analyze the effects of Quaternary glaciations on three species of scrubwren (buff-faced scrubwren, *Aethomyias perspicillatus*; Papuan scrubwren, *A. papuensis*; and large scrubwren, *Sericornis nouhuysi*) endemic to Papua New Guinea that co-inhabit the slopes of its tallest mountain, Mt Wilhelm (4509 m). These three species belong to a large clade of small-sized Papuan passerines; they were formerly classified in a single genus *Sericornis*, but we here adhere to the most updated genus classification following recent research into this radiation’s evolutionary history [16]. All three of these scrubwrens are sedentary in nature and found in (sub-) montane forest at various elevational bands [16–18]. They are mostly insectivorous, with *S. nouhuysi* also known to consume seeds at times [17, 19]. *S. nouhuysi* has the widest elevational tolerance and is sympatric with both *A. perspicillatus*, which is restricted to lower montane forest at 1500–2450 m, and with *A. papuensis*, which is mostly found in upper montane habitat above 2000 m [17].

**Fig. 1:**
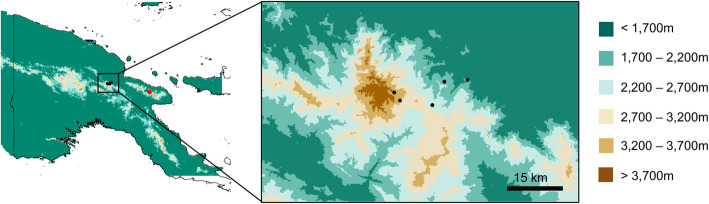
Topographical map of Papua New Guinea indicating the sampling locations in black and red dots; inset shows vicinity of Mt. Wilhelm. Black dots represent the sampling locations on Mt. Wilhelm and the red dot represents the sampling location on the Finisterre range

Using genome-wide data, we investigate the population structure and demographic history of the three scrubwren species across the elevational gradient on Papua New Guinea’s highest mountain, Mount Wilhelm (Fig. 1), specifically to test the hypothesis of whether global cooling associated with the Last Glacial Maximum (LGM) has led to demographic fluctuations or not. Mt. Wilhem has been strongly affected by Quaternary glaciations, and was covered by ice at least until 15,000 years ago during the most recent ice age [20]. Using an approximate Bayesian computational approach, we assess various scenarios of population fluctuation across all three species against paleoclimatological timelines of vegetational change to provide an unprecedented look into the effects of Quaternary climate change on biotic evolution in New Guinea’s mountains. Based on our understanding of Quaternary glaciations, we expect a strong effect of the LGM on all three species, characterized by a possible signature of population expansion during the LGM as each species descended from the mountain and occupied a larger geographic area. These analyses will help shed light on the larger question of how Quaternary climatic oscillations have affected montane tropical biota.

## Results

### Sequence data and phylogenomic reconstruction

We sampled 74 scrubwrens across an elevational gradient in Mt Wilhelm (Fig. 1), Papua New Guinea and generated a genome-wide double digest restriction enzyme associated DNA sequence (ddRADseq) dataset (see the methods section for details). We obtained ~ 104 million cleaned reads from the ddRADseq runs. The average number of reads per sample for ddRADseq data was 1.3 million (± 0.85 million, standard deviation; Table S1). As scrubwrens can be difficult to differentiate in the field [18], we confirmed species identity based on both the *COI* (cytochrome oxidase I gene) gene network and phylogenomic tree (Fig. S1). Species identity based on both *COI* and nuclear data were in agreement.

We observed non-sister relationships among the three focal taxa (Fig. S1A) based on 1040 loci amounting to 142,631 bp of concatenated sequence data in agreement with previous studies [21, 22]. For both *A. papuensis* and *A. perspicillatus* the number of mitochondrial haplotypes was low (Fig. S1B). We observed a star shaped haplotype network for *S. nouhuysi* suggesting recent expansion (Fig. S1B). The pairwise *COI* distance between scrubwren species varied from 7.9 to 13.4% (Table S2).

### Population-genomic summary statistics

Based on the genetic identification of individuals, we generated separate SNP datasets in STACKS for *A. perspicillatus*, *A. papuensis* and *S. nouhuysi*. We obtained over a thousand SNPs for each species and no locus was found to be under selection (Table S3). Increasing the number of differences between individuals at a locus (n = 0, n = 1, n = 2) did not lead to a drastic increase in the number of SNPs obtained (Table S3). Therefore, we used the SNP dataset with the following STACKS parameters: stack depth of at least ten (m = 10); two differences between reads within a stack (M = 2) and a single difference between individuals for a locus (n = 1).

Genetic diversity for *S. nouhuysi* was generally lower as compared to the other two species of scrubwren, both on the basis of unlinked SNP data and sequence alignments (Tables 1 and 2). For all three species, Tajima’s D was negative, suggesting recent population expansion (Tables 1 and 2).

**Table 1:** Summary statistics based on neutral SNP data

Species	Number of neutral SNPs	Effective number of alleles	Observed heterozygosity	Polymorphic information content
*Aethomyias perspicillatus*	1984	1.193	0.132	0.126
*Aethomyias papuensis*	2829	1.196	0.132	0.122
*Sericornis nouhuysi*	1218	1.128	0.089	0.086

**Table 2:** Summary statistics based on filtered RAD-loci (sequence based)

Species	Total sequence length (bp)	Nucleotide diversity (π)	Tajima’s D
*Aethomyias perspicillatus*	278,104	0.0031	−0.967
*Aethomyias papuensis*	396,331	0.0023	−0.729
*Sericornis nouhuysi*	170,658	0.0018	−1.054

### Elevational distribution of species

Our mist-netting activities largely confirmed previous information on the elevational distribution of these three species (e.g., [18]). The two more closely related species (Fig. S1A) were elevationally segregated, with *A. perspicillatus* occupying lower elevations (1700 m to 2200 m) whereas *A. papuensis* occurred at higher elevations (2200 m to 3700 m). The more distantly related *S. nouhuysi* (Fig. S1A) appears ecologically compatible with both other species and co-occurs with them across their entire elevational range (1700 m to 3700 m).

### Lack of population structure

**Fig. 2:**
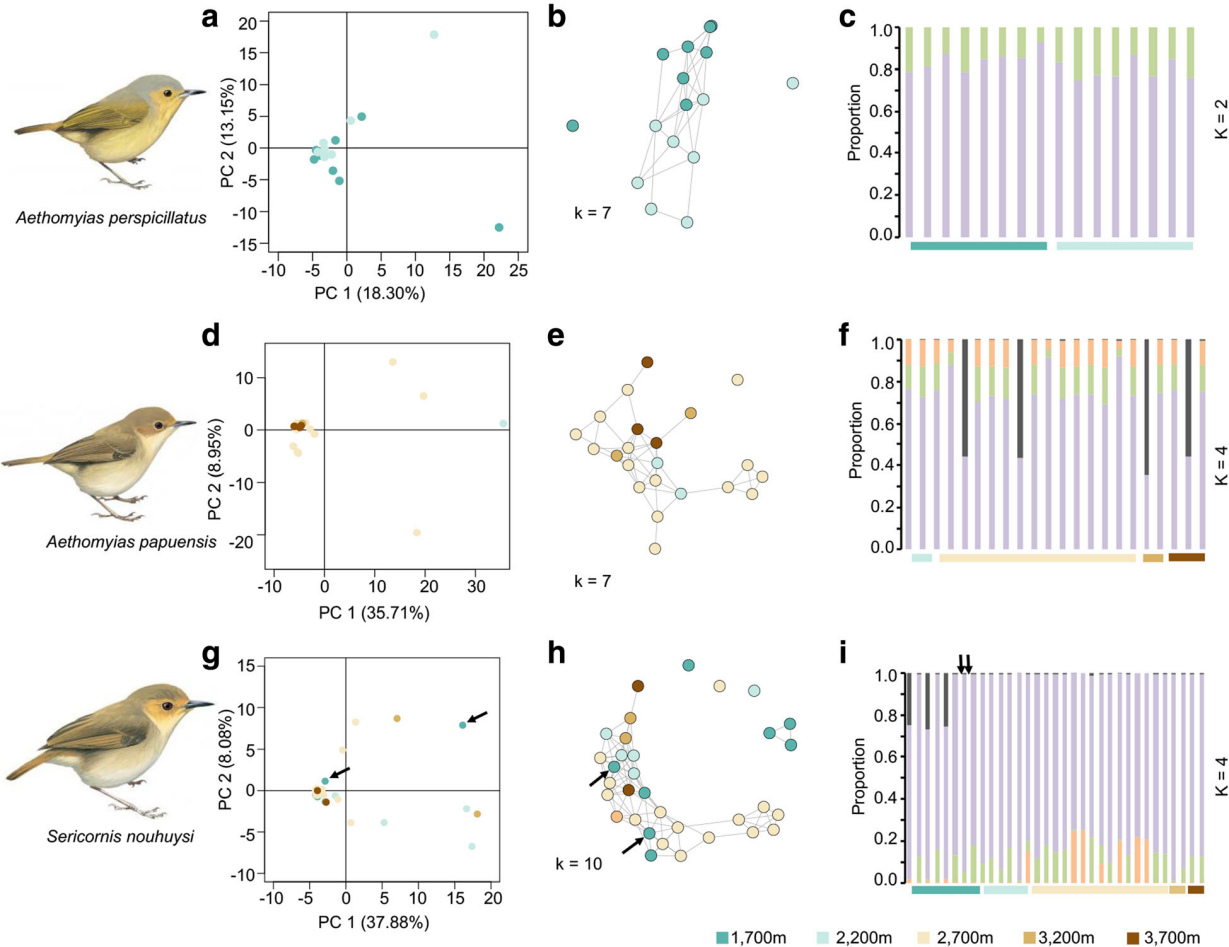
Population-genomic subdivision in the three species of scrubwren based on principal coordinate analysis (left), network-based analysis (middle) and STRUCTURE analysis (right). See Table 1 for the number of SNPs used in each analysis. The two *S. nouhuysi* individuals from the Finisterre Range are marked with arrows. Bird illustrations modified from del Hoyo et al. [23]

Low or no elevational differentiation was detected in the three species of scrubwren based on multiple approaches (principal coordinate analysis (PCoA), network analysis and STRUCTURE) (Fig. 2). We generally observed a single population cluster in PCoA analysis in each of the three species (Fig. 2a, d, g), with minor outliers. Based on Evanno et al.’s [24] method to find the most likely number of clusters (K) in STRUCTURE, K = 2 performed best in *A. perspicillatus* and K = 4 in both *A. papuensis* and *S. nouhuysi* (Fig. S2). However, no elevation-specific pattern of differentiation emerged in both STRUCTURE and similarity-based network analyses (Fig. 2b, c, e, f, h, i). Even the two individuals of *S. nouhuysi* from the Finisterre range, roughly 180 km from Mt. Wilhelm, did not segregate (indicated by black arrows in Fig. 2g, h and i). F_ST_ and Dxy analyses suggested limited, non-significant differentiation among elevational bands in *A. perspicillatus* and *A. papuensis* (Table S4). Only for *S. nouhuysi*, we observed significant subdivision based on pairwise F_ST_ estimates between populations (Table S4C), with a more pronounced differentiation between higher elevations (3200 m to 3700 m) and lower elevations (1700 m to 2700 m). However, no such differentiation was suggested by Dxy analysis (Table S4C). No isolation by distance was detected in any of the three species of scrubwren across the Mt Wilhelm study area (Fig. S3).

### Coalescent modelling

Based on approximate Bayesian computational (ABC) analyses, an evolutionarily recent population expansion (after the LGM; model B; Fig. S4) was the best model for all three species using both simple rejection and regression-based analyses (Table 3). For all three species, we observed an expansion around 27,000 to 29,000 years ago assuming a generation time of 1 year (Table 4). The observed data were within both the prior and posterior space. The error rate for model selection was zero and bias in parameter estimation was low (Table S5).

**Table 3:** Posterior probabilities of demographic models

Model	Species					
	*Aethomyias perspicillatus*		*Aethomyias papuensis*		*Sericornis nouhuysi*	
	Based on rejection	Using logistic approach	Based on rejection	Using logistic approach	Based on rejection	Using logistic approach
Constant population size	0.0	0.0	0.0	0.0	0.0	0.0
Population expansion after last glacial maximum	1.0	1.0	1.0	1.0	1.0	1.0
Population expansion during last interglacial period	0.0	0.0	0.0	0.0	0.0	0.0

**Table 4:** Median parameter values with 5 and 95% quantile values (in brackets) based on the most highly supported model (model B; Fig. S4)

Species	Ancestral effective population size (NA)	Effective population size post-expansion (N)	Time of expansion (TEXP1)
*Aethomyias perspicillatus*	8100 (4040–11,400)	114,000 (57,100–146,000)	29,000 (14,100–38,000)
*Aethomyias papuensis*	15,500 (8010–20,600)	116,000 (60,000–146,000)	29,100 (14,800–37,900)
*Sericornis nouhuysi*	10,800 (5590–15,100)	157,000 (82,600–195,000)	27,100 (13,800–36,800)

**Fig. 3:**
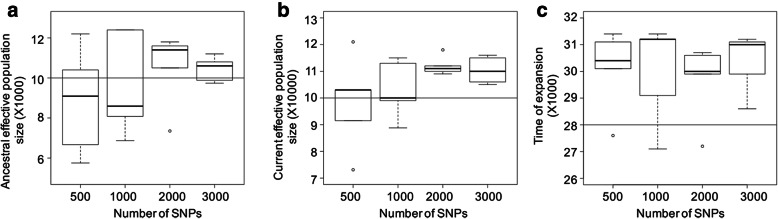
Parameter estimation in DIYABC across four simulated datasets with increasing numbers of SNPs for **a** ancestral effective population size, **b** current effective population size and **c** time of expansion. The horizontal line represents the value used for data simulations (see main text for details)

For the simulated datasets we generally observed low error rates in model identification. For the simulated SNP datasets, the estimated time of expansion was slightly higher than the simulated time of expansion (Fig. 3c). Furthermore, we observed that the standard deviation in parameter estimation decreased with an increase in the number of SNPs (Fig. 3).

## Discussion

### Species identification and phylogenetic reconstruction

While phylogenetic relationships of our target scrubwren species were not the main interest of this study, we did use a phylogenomic approach as a supplementary line of evidence for two reasons: (1) the substantial phenotypic conservatism in these scrubwrens, which share a nearly identical morphology [17, 18], necessitated unequivocal identification of our samples not only on the basis of a mitochondrial barcode (which may be subject to genetic introgression artifacts [25]), but on the basis of genome-wide markers; (2) it was important to ensure that Mt Wilhelm populations of all three lineages are independent members of the scrubwren radiation, preferably in non-sister placements to reduce the possibility of secondary gene flow artifacts.

When we compared morphology-based identifications by our field personnel to genetic identity based on genome-wide markers and mitochondrial sequences (partial *COI*), only 66.2% of field identifications were accurate. Around 33.8% of the samples were misidentified in the field based on their confusing plumage similarity. Mostly *S. nouhuysi* was misidentified as either *A. papuensis* (n = 9) or *A. perspicillatus* (n = 5). Phylogenomic analysis of genome-wide alignments of 1040 loci spanning 142,631 bp confirmed all three species as members of the *Sericornis/Aethomyias* scrubwren radiation (Fig. S1B). They emerged in a non-sister species placement with regards to one another in agreement with previous findings [21]. The observed sympatry in the field is consistent with their non-sister relationships, given that young species pairs tend to be allopatric or parapatric [26].

### Lack of population structure

All three species of scrubwren showed a general lack of population structure (Fig. 2), suggesting high levels of gene flow across the Mt Wilhelm study area and / or a recent population expansion. Only in *S. nouhuysi* did we observe some limited segregation of high elevation individuals (above 3000 m) in F_ST_ values, which, however, was not reflected by Dxy values (Table S4C). This result may be attributable to the relatively low sample number available for this species at high elevations (3200 m, n = 2; 3700 m, n = 2). More importantly, F_ST_ values quickly become inflated when genetic diversity is low [27, 28], as is the case in *S. nouhuysi* (Table S4C) when compared to the other two species. In such cases, Dxy is a better measure of genetic differentiation as it is independent of population-internal genetic diversity [27]. We therefore conclude that population structure of *S. nouhuysi* on Mt. Wilhelm is likely as undifferentiated as in the other two species.

### Population expansion at the onset of the Last Glacial Maximum

All three species of scrubwren exhibited a strong signal of population expansion around 27,000 to 29,000 years ago (Model B, Fig. S4; Tables 3 and 4). Despite differences in body size and elevational occurrence, the congruence of the timing of population expansion among all three species is remarkable. The expansion period falls within the onset of the LGM following the transition out of the Ålesund Interstadial at ~ 30,000 years ago [29], which was marked by the most precipitous fall in global sea level and accompanying drops in global temperature across the most recent ice age. The strong cooling would have shifted elevational belts down the slope, expanding the area of habitat available to montane forest birds such as the three scrubwrens, and sometimes even connecting populations that would have previously been stranded on separate mountain ranges. During the glacial peak, Papua’s alpine and subalpine grasslands descended to 2500 m elevation, leading to a concomitant shift in the distribution of montane forest due to a downward movement of the upper treeline by more than 1200 m during the LGM [30]. Power analysis to test the reliability of ABC results (Fig. 3) provides marginally older estimates of population expansion (~ 30,000 years ago), still well within the onset of the LGM. More importantly, these power analyses underscore that expansion events simulated on the basis of lesser SNP sets than ours reliably estimate the parameters in question. Our study corroborates the utility of this approach, as advocated by Cabrera and Palsbøll [31], and calls for its more widespread utilization.

The over five-fold increase in effective population size at the onset of the LGM suggests a strong demographic response in all three species (Table 4) that is only compatible with a massive synchronous expansion in breeding habitat, perhaps coupled with an influx of alleles from newly-connected populations. For instance, our results suggest limited differentiation even between populations on widely-disjunct mountain ranges, such as *S. nouhuysi* from Mt. Wilhelm and the Finisterre Range (Fig. 2), bearing testimony of their recent connection during the LGM.

### The role of global cooling cycles in tropical montane biotic evolution

Much is known about the LGM’s role in fragmenting populations, especially at higher latitudes [2–4, 32], but we are only beginning to understand the opposite effect that global cooling cycles have had on many tropical montane biota (e.g. [14]). Ours is one of the first studies based on genome-wide loci, certainly for the Australasian tropics, to demonstrate the important role of global cooling cycles in triggering population expansions of tropical montane vertebrates. Conversely, warmer interglacials are expected to have the opposite impact, fragmenting montane tropical fauna on mountaintops into isolated populations. It is instructive to see that our data do not show an obvious signature of a recent decline in population genetic diversity consistent with the end of the LGM, most likely because of a lag time for genetic declines to manifest themselves and the recency of our current interglacial period, which only started roughly 10,000 years ago.

## Conclusion

The Quaternary has had a strong impact on current global biodiversity patterns. We observed a strong signal of population expansions at the onset of the LGM across three montane scrubwrens in Papua New Guinea. Global cooling would have led to a downward shift and increase in suitable habitats in these species, all of which inhabit montane forest. Despite differences in body size and elevational occurrence all three species of scrubwren show concordant signals of population expansion around 27,000 to 29,000 years ago. Our study suggests that global cooling periods may have had opposing effects on tropical mountains versus temperate and boreal latitudes, where most species underwent serious fragmentation rather than expansion during glaciations.

## Methods

### Sampling and DNA extraction

We mist-netted birds along an elevational gradient on Mt. Wilhelm (from 200 m to 3700 m; see Sam and Koane [33] for sampling details; see Fig. 1 and Table S1 for locality and specimen information) in Papua New Guinea. We sampled every 500 m elevational interval, collected blood from 74 captured scrubwren individuals through brachial venipuncture and stored it in 95% ethanol prior to DNA extraction (Table S1). Individuals were tagged and released within ten minutes [33]. In addition, we sampled two individuals from the Finisterre range (for locality information see Fig. 1 and Table S1). All individuals were identified based on external morphology, but given the difficulties associated with morphological identification in this group [18], we supplemented our field identification with genomic data (see below). Based on morphological identification we captured Papuan scrubwrens *A. papuensis* (n = 26), buff-faced scrubwrens *A. perspicillatus* (n = 23), large scrubwrens *S. nouhuysi* (n = 24), pale-billed scrubwrens *A. spilodera* (n = 2) and grey-green scrubwrens *A. arfakianus* (n = 1). We also sampled two individuals of the mountain mouse-warbler *Origma robusta* from Mt. Wilhelm as an outgroup for some of our analyses. In addition to our own sampling efforts, we obtained tissue samples for yellow-throated scrubwren *Neosericornis citreogularis* (n = 2); white-browed scrubwren *S. frontalis* (n = 1); large-billed scrubwren *S. magnirostis* (n = 1) and pale-billed scrubwren *A. spilodera* (n = 1) from the Burke Museum of Natural History and Culture (for locality information, see Table S1).

We used the DNeasy Blood and Tissue Kit (QIAGEN, Germany) to extract genomic DNA following the manufacturer’s instructions and quantified DNA using a Qubit 2.0 high sensitivity DNA Assay kit (Invitrogen, USA).

### Ethics statement

This study complied with all ethical regulations and protocols were approved by the National University of Singapore Institutional Animal Care and Use Committee (IACUC, Protocol Number: L2017–00459).

### ddRADseq library preparation and Illumina sequencing

We prepared ddRADseq libraries using established protocols with modifications [34, 35]. We used a 6 bp (EcoRI-HF) and a 4 bp (MspI) restriction enzyme to digest the DNA, and used Sera-Mag magnetic beads (Thermo Scientific, USA) for size selection (300-500 bp fragments). Twelve PCR cycles were performed for final library preparation and samples were pooled at equimolar concentrations. A quality check for the final pooled libraries was performed using a Fragment Analyser (Advanced Analytical). We carried out two paired-end runs of 150 bp on an Illumina HiSeq 2500 with a 5% PhiX spike to avoid low sequence diversity issues.

### ddRADseq data filtering and data matrix generation

We performed a visual quality check for the raw data using FASTQC v.0.11.5 [36]. Raw reads were then filtered and demultiplexed using the process_radtags program within the STACKS 1.44 [37] pipeline. We trimmed the reads to 140 bp and removed all reads that contained at least one uncalled base or were otherwise of low quality (phred score < 10). During demultiplexing we allowed for one mismatch in barcodes. Demultiplexed data were used for both phylogenomic and population genomic analyses. Given the recovery of thousands of loci, we used only read 1 across all analyses to avoid the inclusion of SNPs in linkage disequilibrium. We used two different pipelines for data processing. For phylogenomic analyses based on concatenated sequence data, we employed the pyRAD 3.0.66 pipeline [38] and for population genomic analyses based on genome-wide SNPs, we used the STACKS pipeline.

For phylogenomic data generation, we applied default settings within pyRAD while keeping the minimum locus coverage to six reads, allowing for a maximum of four bases within a read with a phred score of < 20, and using a clustering threshold of 0.88 for both within and between samples. We allowed for a maximum of 30% missing data and generated a concatenated sequence matrix. All samples were included for this analysis.

In our population-genomic analyses, we generated separate SNP datasets for the three species of scrubwren (*A. perspicillatus*, *A. papuensis* and *S. nouhuysi*). The denovo_map.pl program within STACKS was used for SNP calling. We set the minimum stack depth to ten (m = 10) and further allowed for two mismatches (M = 2) between stacks within an individual. We varied the number of differences between loci during catalog building from zero to two (n = 0, 1 and 2) and removed highly repetitive stacks suggestive of paralogs. Default settings were applied in STACKS for SNP calling and SNPs were filtered using the ‘populations’ program. We allowed for a maximum of 20% missing data per locus and called only a single random SNP per locus.

### Mitochondrial DNA barcoding

We amplified 313 bp of the mitochondrial *COI* gene [39] using universal metazoan primers [40, 41] with a 9 bp barcode which differed across samples in at least 4 bases. The following PCR conditions were used: initial denaturation at 94 °C for five minutes, denaturation at 94 °C for 30 s, annealing at 48 °C and extension at 72 °C for a minute each for 35 cycles. Final extension was carried out at 72 °C for five minutes. Samples were pooled at equal concentrations, cleaned using SureClean (Bioline Inc., London) following the manufacturer’s instructions, and sequenced on a MiSeq platform using a 300 bp paired-end run. Sequences were edited and assembled following Meier et al. [39] and samples with at least 10X coverage were used for further analyses. Based on these criteria, we were able to successfully amplify the *COI* gene fragment for 75 samples (Table S1).

In addition to the sequences generated in this study, we also included *COI* sequences for other scrubwrens from GenBank (Table S6). We performed multiple *COI* sequence alignments using MAFFT [42]. A phylogenetic haplotype network was constructed using the TCS method [43] in PopART 1.7 [44]. Pairwise net p-distances between species were computed in MEGA 7 [45].

### Phylogenomic analysis

On the basis of our concatenated pyRAD data matrix, we reconstructed phylogenetic relationships using the maximum likelihood framework implemented in RAxML GUI 1.5 [46]. We used the GTR + Gamma model of sequence evolution and performed a single full maximum likelihood tree search, employing the rapid bootstrap algorithm with 1000 replicates. The final tree was rooted using *Origma robusta* following Marki et al. [21] and visualized in FigTree 1.4.2 [47].

### Population genomic analysis

Three species-specific SNP datasets obtained from STACKS were used for downstream analyses. We first filtered our data and removed any non-neutral loci using BAYESCAN 2.1 [48]. Then, for the filtered dataset, we estimated the level of missing data in PLINK 1.0.9 [49] and calculated summary statistics in GENODIVE 2.0b27 (effective number of alleles and observed heterozygosity) [50] and CERVUS 3.0.7 (polymorphic information content) [51, 52]. Further, for the filtered loci, we estimated nucleotide diversity and Tajima’s D in DnaSP 6 [53] using the strict fasta file obtained from STACKS.

#### Population subdivision

We used multiple approaches to characterize population structure in each lineage as a baseline for further analyses. Firstly, on the basis of the filtered SNPs, we computed pairwise F_ST_ values in Arlequin 3.5.2.2 [54] using the distance method, and calculated Dxy (average number of nucleotide substitutions per site between populations) using the entire RAD-locus sequences for the same filtered loci in DnaSP, grouping individuals based on sampled elevational bands. For the F_ST_ estimates, we performed 10,000 permutations to test for significant differentiation. We ran a PCoA using GenAlEx [55] and plotted results in R 3.2.4 [56]. PCoA is a multidimensional scaling approach to visually represent similarity or differences among individuals. To perform PCoA, we estimated Nei’s genetic distance D [57, 58] based on SNP data in GenAlEx.

In addition to PCoA, we employed the ‘netview’ package [59] in R to analyse genetic structure using mutual k-nearest neighbour graphs. Netview utilizes network theory principles to identify fine-scale subdivision [59]. We applied the genetic distances estimated in PLINK to construct networks and used multiple approaches (Fast-Greedy, Infomap and Walktrap) to determine the best ‘k’ for network analysis, with ‘k’ being defined as the maximum number of mutual nearest neighbours that can be connected by edges during network construction [59]. Following this approach, we plotted out network for different values of k.

Finally, we used the Bayesian clustering approach in STRUCTURE 2.3.4 [60] to infer the number of genetic clusters (‘K’) within the data. We performed ten iterations each for K = 1 to K = 8. For each iteration, we performed 100,000 generations of burnin and 500,000 generations of MCMC sampling. We used STRUCTURE HARVESTER [61] and the delta K method [24] to infer the optimal number of clusters (K) and compared results across different K values.

#### Isolation by distance

Scrubwrens’ extremely sedentary lifestyle makes them susceptible to breaks in gene flow across barriers in topographically diverse areas such as Mt Wilhelm, even at spatial scales spanning only dozens of kilometers. Therefore, we tested for isolation by distance in GenAlEx by comparing Nei’s genetic distance D and geographic distance. An individual-based Mantel test with 999 bootstraps was performed to test for significant associations between genetic and geographic distance. For *S. nouhuysi*, we removed the two disjunct samples from the Finisterre range in these tests.

#### Coalescent modelling

We adopted an ABC approach as implemented in DIYABC v2.1.0 [62] to understand the demographic history of the three species of scrubwren. Preliminary analyses indicated that all three species have gone through an evolutionarily recent population expansion. To explore whether this expansion would have been associated with glacial periods of colder temperatures and descending elevational bands or with warmer interglacials comparable to present-day climatic conditions, we explored three different demographic models for each species: constant population size, population expansion during the last glaciation (~ 40,000 to 10,000 years ago) and population expansion during the last interglacial period (~ 130,000–110,000 years ago) (Fig. S4). Population contractions during the LGM or prior had been taken into account and ruled out in preliminary, exploratory ABC runs (not shown), which is why we concentrated on testing population expansion versus stability and differentiating among multiple scenarios of expansion. We used uniform prior distributions for all parameters (Table S7) and performed one million simulations for each model. As with the isolation by distance analyses, we removed the two samples from the Finisterre range for *S. nouhuysi* and modelled demographic expansion. We included the high elevation populations of *S. nouhuysi* in our demographic analysis despite significant subdivision based on F_ST_, as the sample size for high elevation populations was low and the subdivision was not significant based on Dxy estimates (see results section).

We used both rejection and linear regression methods for model selection based on four summary statistics (Table S8). For the best model, we confirmed that the observed data were within the posterior space. To test the power of the data to differentiate among models, we calculated the posterior error rate in DIYABC using pseudo-observed data. We generated pseudo-observed datasets by sampling (with replacement) a specific model and associated parameter values from the 500 simulations that are closest to the observed data. We calculated the posterior probability for each pseudo-observed dataset and assessed the proportion of times the correct model has the highest probability. Further, we estimated demographic parameters for the best model and generated 500 datasets based on the posterior distribution to estimate the bias and precision in parameter estimation. We assumed a generation time of 1 year for all three species, which is a widespread estimate for the generation time of such small-sized passerines [63].

To test the power of DIYABC to accurately identify the correct model and estimate parameters, we also simulated SNP datasets in DIYABC assuming an effective population expansion from 10,000 to 100,000 individuals around 28,000 years ago. To test the effect of the number of SNPs on the accuracy of parameter estimation in DIYABC, we varied this number from 500 to 3000 (number of SNPs: 500; 1000; 2000 and 3000) and generated five different datasets for each SNP setting. Thus, in total we generated 20 different datasets. For each dataset we sampled 20 individuals (ten males and ten females). We then ran the above three demographic history scenarios for each simulated dataset and estimated parameters for the best model.

## Supplementary information

**Additional file 1 Figure S1.** A) Phylogenomic analysis of a concatenated alignment of 1040 genomic loci totaling 142,631 bp using RAxML, with asterisks indicating nodal bootstrap support values equal to or above 85 for key nodes; B) haplotype network using the TCS method as implemented in PopArt based on 313 bp of the *COI* gene.

**Additional file 2 Figure S2.** Selection of best K for Structure analysis following Evanno et al.’s [24] method; A) *Aethomyias perspicillatus*, B) *Aethomyias papuensis*, and C) *Sericornis nouhuysi*.

**Additional file 3 Figure S3.** Isolation by distance graphs, plotting Nei’s genetic distance versus geographical distance for pairwise comparisons within A) *Aethomyias perspicillatus*, B) *Aethomyias papuensis*, and C) *Sericornis nouhuysi*.

**Additional file 4 Figure S4.** Models simulated for DIYABC analysis. See Table S7 for more details. Nc: Effective population size in constant model; N: Effective population size post expansion; NA: Ancestral effective population size; TEXP1 and TEXP2: Time of expansion.

**Additional file 5 Table S1.** Details of samples used in this study along with the number of cleaned reads obtained from Illumina sequencing and details on *COI* gene amplification. Museum abbreviation: BMNHC = Burke Museum of Natural History and Culture, Seattle, USA.

**Additional file 6 Table S2.** Genetic pairwise *COI* distances (p distances) between various scrubwren species are shown below the diagonal and standard error estimates are shown above the diagonal.

**Additional file 7 Table S3.** Number of SNPs identified by increasing the number of differences between individuals at a locus in STACKS. **Table S4.** Pairwise F_ST_ estimates between elevational bands for A) *Aethomyias perspicillatus* B) *Aethomyias papuensis* and C) *Sericornis nouhuysi.* Values in the lower left triangle refer to pairwise F_ST_ estimated using the distance method in Arlequin [54]; in the upper right triangle they refer to pairwise Dxy values (average number of nucleotide substitutions per site between populations) estimated using DnaSP [53]. Significant F_ST_ values are indicated in bold font. **Table S5.** Mean relative bias for parameter estimation in DIYABC for the most supported model. **Table S6.** Accession numbers of Genbank samples (not generated by us) used in *COI* gene network analysis. **Table S7.** Priors used for coalescent modelling in DIYABC. See Fig. S4 for the models. **Table S8.** Summary statistics used for DIYABC analyses.

## Data Availability

The ddRAD-Seq data (Sequence Read Archive: SRP158801) and COI gene sequences (MK507157-MK507229) generated in this study are available on NCBI.

## Abbreviations

LGM: Last Glacial Maximum
ddRADseq: double digest restriction enzyme associated DNA sequence
*COI*: Cytochrome oxidase I gene
PCoA: Principal coordinate analysis
ABC: Approximate Bayesian computational

## References

[1] Bintanja R, van de Wal RS, Oerlemans J (2005). Modelled atmospheric temperatures and global sea levels over the past million years. Nature.

[2] Hewitt G (2000). The genetic legacy of the quaternary ice ages. Nature..

[3] Hewitt G (2004). Genetic consequences of climatic oscillations in the quaternary. Philos Trans R Soc B.

[4] Seddon J, Santucci F, Reeve N, Hewitt G (2001). DNA footprints of European hedgehogs, *Erinaceus europaeus* and *E. concolor*: Pleistocene refugia, postglacial expansion and colonization routes. Mol Ecol.

[5] Taberlet P, Fumagalli L, Wust-Saucy AG, Cosson JF (1998). Comparative phylogeography and postglacial colonization routes in Europe. Mol Ecol.

[6] Weigelt P, Steinbauer MJ, Cabral JS, Kreft H (2016). Late quaternary climate change shapes island biodiversity. Nature..

[7] Garg KM, Chattopadhyay B, Wilton PR, Prawiradilaga DM, Rheindt FE (2018). Pleistocene land bridges act as semipermeable agents of avian gene flow in Wallacea. Mol Phylogenet Evol.

[8] Ng NS, Wilton PR, Prawiradilaga DM, Tay YC, Indrawan M, Garg KM, Rheindt FE (2017). The effects of Pleistocene climate change on biotic differentiation in a montane songbird clade from Wallacea. Mol Phylogenet Evol.

[9] Kershaw A, Van Der Kaars S, Flenley J. The quaternary history of far eastern rainforests. In: Tropical rainforest responses to climatic change. Berlin: Springer; 2011. p. 85–123.

[10] Qu Y, Luo X, Zhang R, Song G, Zou F, Lei F (2011). Lineage diversification and historical demography of a montane bird *Garrulax elliotii*-implications for the Pleistocene evolutionary history of the eastern Himalayas. BMC Evol Biol.

[11] Gao Y-D, Zhang Y, Gao X-F, Zhu Z-M (2015). Pleistocene glaciations, demographic expansion and subsequent isolation promoted morphological heterogeneity: a phylogeographic study of the alpine *Rosa sericea* complex (Rosaceae). Sci Rep.

[12] Shepard DB, Burbrink FT (2009). Phylogeographic and demographic effects of Pleistocene climatic fluctuations in a montane salamander, *Plethodon fourchensis*. Mol Ecol.

[13] Cabanne GS, Calderón L, Trujillo Arias N, Flores P, Pessoa R, d'Horta FM, Miyaki CY (2016). Effects of Pleistocene climate changes on species ranges and evolutionary processes in the Neotropical Atlantic Forest. Biol J Linn Soc.

[14] Chattopadhyay B, Garg KM, Gwee CY, Edwards SV, Rheindt FE (2017). Gene flow during glacial habitat shifts facilitates character displacement in a Neotropical flycatcher radiation. BMC Evol Biol.

[15] Capurucho JMG, Ashley MV, Ribas CC, Bates JM (2018). Connecting Amazonian, Cerrado, and Atlantic forest histories: Paraphyly, old divergences, and modern population dynamics in tyrant-manakins (*Neopelma*/*Tyranneutes*, Aves: Pipridae). Mol Phylogenet Evol.

[16] Norman JA, Christidis L, Schodde R (2018). Ecological and evolutionary diversification in the Australo-Papuan scrubwrens (*Sericornis*) and mouse-warblers (*Crateroscelis*), with a revision of the subfamily Sericornithinae (Aves: Passeriformes: Acanthizidae). Org Divers Evol.

[17] Gregory P, del Hoyo J, Elliott A, Sargatal J, Christie DA, de Juana E (2018). Genus *Sericornis*. Handbook of the birds of the world alive.

[18] Pratt TK, Beehler BM (2014). Birds of New Guinea.

[19] Sam K, Koane B, Jeppy S, Sykorova J, Novotny V (2017). Diet of land birds along an elevational gradient in Papua New Guinea. Sci Rep.

[20] Mills S, Barrows T, Hope G, Pillans B, Fifield K (2016). The timing of late Pleistocene glaciation at mount Wilhelm, Papua New Guinea. EGU General Assembly Conference Abstracts.

[21] Marki PZ, Jønsson KA, Irestedt M, Nguyen JM, Rahbek C (2017). Fjeldså J Supermatrix phylogeny and biogeography of the Australasian Meliphagides radiation (Aves: Passeriformes). Mol Phylogenet Evol.

[22] Norman JA, Rheindt FE, Rowe DL, Christidis L (2007). Speciation dynamics in the Australo-Papuan Meliphaga honeyeaters. Mol Phylogenet Evol.

[23] del Hoyo J, Elliott A, Sargatal J, Christie D, de Juana E (2018). Handbook of the birds of the world alive.

[24] Evanno G, Regnaut S, Goudet J (2005). Detecting the number of clusters of individuals using the software STRUCTURE: a simulation study. Mol Ecol.

[25] Rheindt FE, Edwards SV (2011). Genetic introgression: an integral but neglected component of speciation in birds. Auk..

[26] Mayr E (1942). Systematics and the origin of species: from the viewpoint of a zoologist.

[27] Cruickshank TE, Hahn MW (2014). Reanalysis suggests that genomic islands of speciation are due to reduced diversity, not reduced gene flow. Mol Ecol.

[28] Delmore KE, Lugo Ramos JS, Van Doren BM, Lundberg M, Bensch S, Irwin DE, Liedvogel M (2018). Comparative analysis examining patterns of genomic differentiation across multiple episodes of population divergence in birds. Evol Lett.

[29] Lambeck K, Rouby H, Purcell A, Sun Y, Sambridge M (2014). Sea level and global ice volumes from the last glacial maximum to the Holocene. Proc Natl Acad Sci U S A.

[30] Hope GS (1976). The vegetational history of Mt Wilhelm, Papua New Guinea. J Ecol.

[31] Cabrera AA, Palsbøll PJ (2017). Inferring past demographic changes from contemporary genetic data: a simulation-based evaluation of the ABC methods implemented in diyabc. Mol Ecol Resour.

[32] Kotlík P, Deffontaine V, Mascheretti S, Zima J, Michaux JR (2006). Searle JB a northern glacial refugium for bank voles (*Clethrionomys glareolus*). Proc Natl Acad Sci U S A.

[33] Sam K, Koane B. New avian records along the elevational gradient of Mt. Wilhelm, Papua New Guinea. Bull Br Ornithol Club. 2014;134:116–33.

[34] Peterson BK, Weber JN, Kay EH, Fisher HS, Hoekstra HE (2012). Double digest RADseq: an inexpensive method for de novo SNP discovery and genotyping in model and non-model species. PLoS One.

[35] Tay Y, Chng M, Sew W, Rheindt F, Tun K, Meier R (2016). Beyond the coral triangle: high genetic diversity and near panmixia in Singapore's populations of the broadcast spawning sea star *Protoreaster nodosus*. R Soc Open Sci.

[36] Andrews S (2010). FastQC: a quality control tool for high throughput sequence data.

[37] Catchen J, Hohenlohe PA, Bassham S, Amores A, Cresko WA (2013). Stacks: an analysis tool set for population genomics. Mol Ecol.

[38] Eaton DA (2014). PyRAD: assembly of de novo RADseq loci for phylogenetic analyses. Bioinformatics.

[39] Meier R, Wong W, Srivathsan A, Foo M (2016). $1 DNA barcodes for reconstructing complex phenomes and finding rare species in specimen-rich samples. Cladistics..

[40] Geller J, Meyer C, Parker M, Hawk H (2013). Redesign of PCR primers for mitochondrial cytochrome c oxidase subunit I for marine invertebrates and application in all-taxa biotic surveys. Mol Ecol Resour.

[41] Leray M, Yang JY, Meyer CP, Mills SC, Agudelo N, Ranwez V, Boehm JT, Machida RJ (2013). A new versatile primer set targeting a short fragment of the mitochondrial COI region for metabarcoding metazoan diversity: application for characterizing coral reef fish gut contents. Front Zool.

[42] Katoh K, Misawa K, Ki K, Miyata T (2002). MAFFT: a novel method for rapid multiple sequence alignment based on fast Fourier transform. Nucleic Acids Res.

[43] Clement M, Posada D, Crandall KA (2000). TCS: a computer program to estimate gene genealogies. Mol Ecol.

[44] Leigh JW, Bryant D (2015). Popart: full-feature software for haplotype network construction. Methods Ecol Evol.

[45] Kumar S, Stecher G, Tamura K (2016). MEGA7: molecular evolutionary genetics analysis version 7.0 for bigger datasets. Mol Biol Evol.

[46] Silvestro D, Michalak I (2012). raxmlGUI: a graphical front-end for RAxML. Org Divers Evol.

[47] Rambaut A (2014). FigTree v1. 4.2: Tree figure drawing tool.

[48] Foll M, Gaggiotti O (2008). A genome-scan method to identify selected loci appropriate for both dominant and codominant markers: a Bayesian perspective. Genetics..

[49] Purcell S, Neale B, Todd-Brown K, Thomas L, Ferreira MA, Bender D, Maller J, Sklar P, De Bakker PI, Daly MJ (2007). PLINK: a tool set for whole-genome association and population-based linkage analyses. Am J Hum Genet.

[50] Meirmans PG, Van Tienderen PH (2004). GENOTYPE and GENODIVE: two programs for the analysis of genetic diversity of asexual organisms. Mol Ecol Notes.

[51] Kalinowski ST, Taper ML, Marshall TC (2007). Revising how the computer program CERVUS accommodates genotyping error increases success in paternity assignment. Mol Ecol.

[52] Marshall T, Slate J, Kruuk L, Pemberton J (1998). Statistical confidence for likelihood-based paternity inference in natural populations. Mol Ecol.

[53] Rozas J, Ferrer-Mata A, Sánchez-DelBarrio JC, Guirao-Rico S, Librado P, Ramos-Onsins SE, Sánchez-Gracia A (2017). DnaSP 6: DNA sequence polymorphism analysis of large data sets. Mol Biol Evol.

[54] Excoffier L, Lischer HE (2010). Arlequin suite ver 3.5: a new series of programs to perform population genetics analyses under Linux and windows. Mol Ecol Resour.

[55] Peakall R, Smouse PE (2012). GENALEX 6.5: genetic analysis in excel. Population genetic software for teaching and research - an update. Bioinformatics..

[56] R Core Team (2016). R: A language and environment for statistical computing.

[57] Nei M (1972). Genetic distance between populations. Am Nat.

[58] Nei M (1978). Estimation of average heterozygosity and genetic distance from a small number of individuals. Genetics..

[59] Neuditschko M, Khatkar MS, Raadsma HW (2012). NETVIEW: a high-definition network-visualization approach to detect fine-scale population structures from genome-wide patterns of variation. PLoS One.

[60] Pritchard JK, Stephens M, Rosenberg NA, Donnelly P (2000). Association mapping in structured populations. Am J Hum Genet.

[61] Earl D (2010). Structure Harvester vO. 56.4.

[62] Cornuet J-M, Pudlo P, Veyssier J, Dehne-Garcia A, Gautier M, Leblois R, Marin J-M, Estoup A (2014). DIYABC v2. 0: a software to make approximate Bayesian computation inferences about population history using single nucleotide polymorphism, DNA sequence and microsatellite data. Bioinformatics..

[63] Cuervo JJ, Møller AP (2017). Colonial, more widely distributed and less abundant bird species undergo wider population fluctuations independent of their popultion trend. PLoS One.

