# Supplementary figures and images for: Last Glacial Maximum led to community-wide population expansion in a montane songbird radiation in highland Papua New Guinea
Source: BMC Evol Biol. 2020 Jul 11;20:82. doi: 10.1186/s12862-020-01646-z (PMC7353695; doi:10.1186/s12862-020-01646-z)

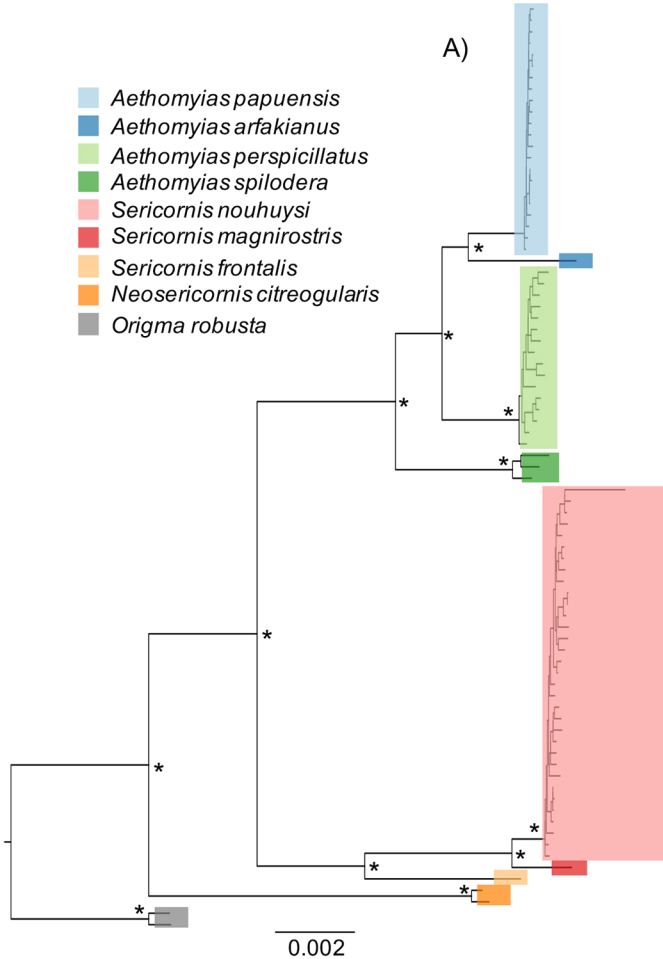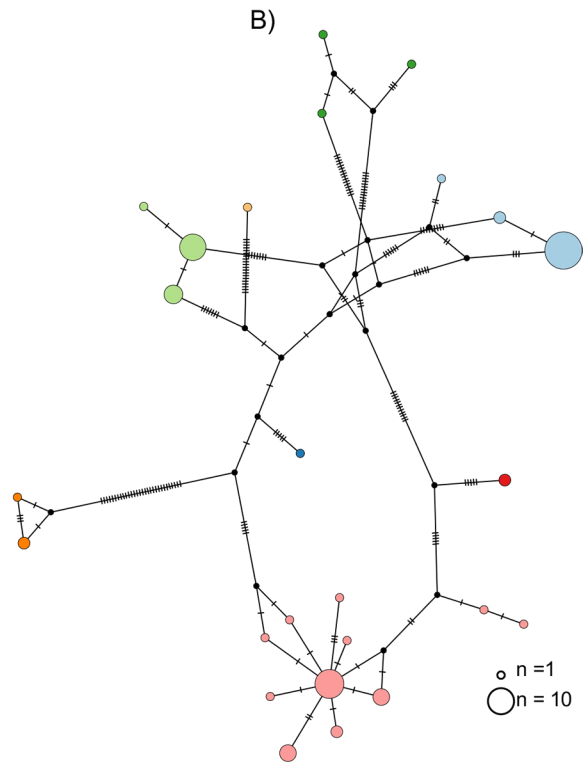

Supplement: Supplementary file 1 — Additional file 1 Figure S1. A) Phylogenomic analysis of a concatenated alignment of 1040 genomic loci totaling 142,631 bp using RAxML, with asterisks indicating nodal bootstrap support values equal to or above 85 for key nodes; B) haplotype network using the TCS method as implemented in PopArt based on 313 bp of the COI gene. [file 12862_2020_1646_MOESM1_ESM.pdf]

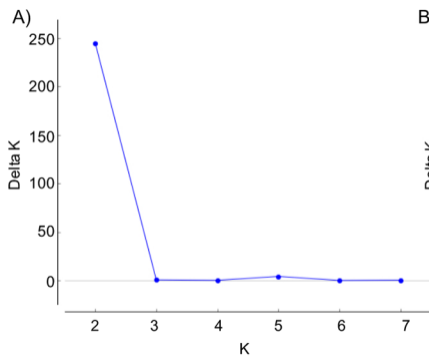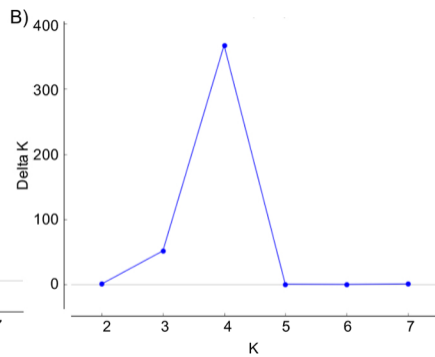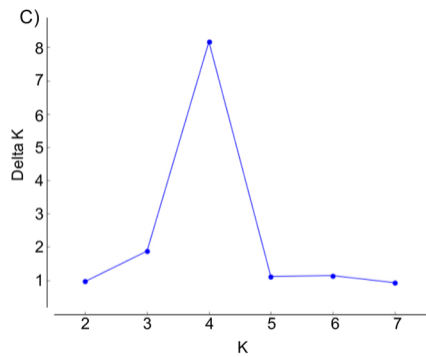

Supplement: Supplementary file 2 — Additional file 2 Figure S2. Selection of best K for Structure analysis following Evanno et al.’s [24] method; A) Aethomyias perspicillatus, B) Aethomyias papuensis, and C) Sericornis nouhuysi. [file 12862_2020_1646_MOESM2_ESM.pdf]

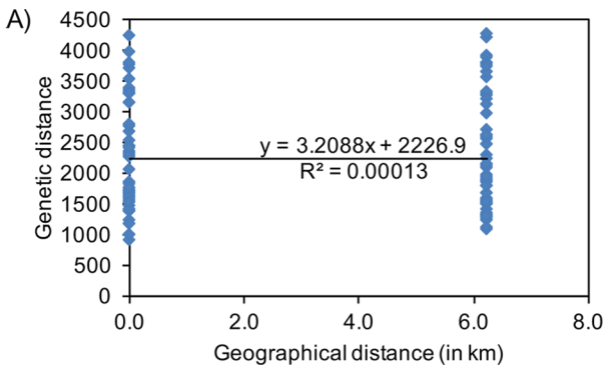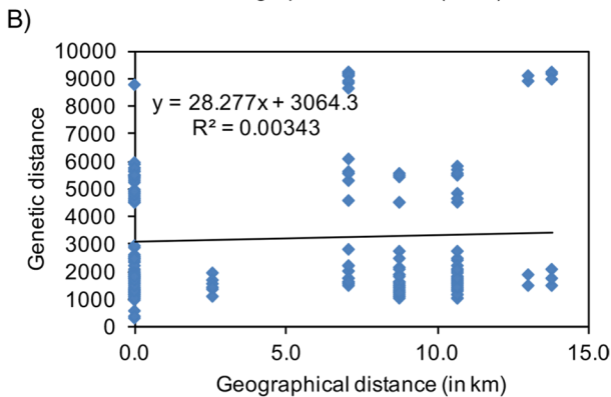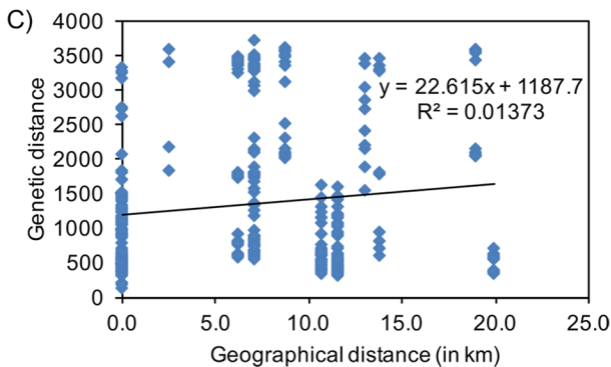

Supplement: Supplementary file 3 — Additional file 3 Figure S3. Isolation by distance graphs, plotting Nei’s genetic distance versus geographical distance for pairwise comparisons within A) Aethomyias perspicillatus, B) Aethomyias papuensis, and C) Sericornis nouhuysi. [file 12862_2020_1646_MOESM3_ESM.pdf]

Past

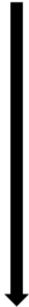

Present

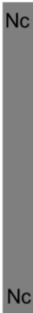

Model A

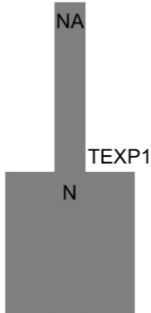

Model B

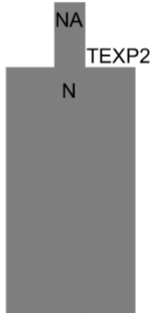

Model C

Supplement: Supplementary file 4 — Additional file 4 Figure S4. Models simulated for DIYABC analysis. See Table S7 for more details. Nc: Effective population size in constant model; N: Effective population size post expansion; NA: Ancestral effective population size; TEXP1 and TEXP2: Time of expansion. [file 12862_2020_1646_MOESM4_ESM.pdf]
