# Supplementary material for: Last Glacial Maximum led to community-wide population expansion in a montane songbird radiation in highland Papua New Guinea
Source: BMC Evol Biol. 2020 Jul 11;20:82. doi: 10.1186/s12862-020-01646-z (PMC7353695; doi:10.1186/s12862-020-01646-z)
Supplement: Supplementary file 7 — Additional file 7 Table S3. Number of SNPs identified by increasing the number of differences between individuals at a locus in STACKS. Table S4. Pairwise FST estimates between elevational bands for A) Aethomyias perspicillatus B) Aethomyias papuensis and C) Sericornis nouhuysi. Values in the lower left triangle refer to pairwise FST estimated using the distance method in Arlequin [54]; in the upper right triangle they refer to pairwise Dxy values (average number of nucleotide substitutions per site between populations) estimated using DnaSP [53]. Significant FST values are indicated in bold font. Table S5. Mean relative bias for parameter estimation in DIYABC for the most supported model. Table S6. Accession numbers of Genbank samples (not generated by us) used in COI gene network analysis. Table S7. Priors used for coalescent modelling in DIYABC. See Fig. S4 for the models. Table S8. Summary statistics used for DIYABC analyses. [file 12862_2020_1646_MOESM7_ESM.docx]

Table S1: Details of samples used in this study along with the number of cleaned reads obtained from Illumina sequencing and details on *COI* gene amplification. Museum abbreviations: BMNHC = Burke Museum of Natural History and Culture, Seattle, USA. (Excel sheet)

Table S2: Genetic pairwise *COI* distances (p distances) between various scrubwren species show below the diagonal and standard error estimate are shown above the diagonal. (Excel sheet)

Table S3: Number of SNPs identified by increasing the number of differences between individuals at a locus in STACKS.

| Species | Number of differences between individuals at a locus (n) | | |
| --- | --- | --- | --- |
|  | n = 0 | n = 1 | n = 2 |
| *Aethomyias perspicillatus* | 1534 | 1984 | 2002 |
| *Aethomyias papuensis* | 2379 | 2829 | 2874 |
| *Sericornis nouhuysi* | 1002 | 1218 | 1184 |

Table S4: Pairwise F_ST_ estimates between elevational bands for method A) *Aethomyias perspicillatus* B) *Aethomyias papuensis* and C) *Sericornis nouhuysi.* Values in the lower left triangle refer to pairwise F_ST_ estimated using the distance method in Arlequin [53]; in the upper right triangle they refer to pairwise Dxy values (average number of nucleotide substitutions per site between populations) estimated using DnaSP [52]. Significant F_ST_ values are indicated in bold font.

Table S4A

|  | 1,700m population | 2,200m population |
| --- | --- | --- |
| 1,700m population (n = 8) | * | 0.0031 |
| 2,200m population (n = 8) | 0.027 | * |

Table S4B

|  | 2,200m population | 2,700m population | 3,200m population | 3,700m population |
| --- | --- | --- | --- | --- |
| 2,200m population (n = 2) | * | 0.0023 | 0.0024 | 0.0024 |
| 2,700m population (n = 15) | -0.021 | * | 0.0024 | 0.0024 |
| 3,200m population (n = 2) | 0.062 | 0.045 | * | 0.0025 |
| 3,700m population (n = 3) | -0.838 | 0.045 | 0.044 | * |

Table S4C

|  | 1,700m population | 2,200m population | 2,700m population | 3,200m population | 3,700m population |
| --- | --- | --- | --- | --- | --- |
| 1,700m population (n = 8) | * | 0.0018 | 0.0019 | 0.0015 | 0.0019 |
| 2,200m population (n = 5) | 0.000 | * | 0.0017 | 0.0014 | 0.0017 |
| 2,700m population (n = 16) | 0.000 | 0.000 | * | 0.0015 | 0.0019 |
| 3,200m population (n = 2) | **0.868** | 0.812 | **0.927** | * | 0.0011 |
| 3,700m population (n = 2) | **0.677** | **0.570** | **0.804** | -0.667 | * |

Table S5: Mean relative bias for parameter estimation in DIYABC for the most highly supported model.

| Species | Ancestral effective population size | Current effective population size | Time of expansion |
| --- | --- | --- | --- |
| *Aethomyias perspicillatus* | 0.090 | 0.117 | 0.109 |
| *Aethomyias papuensis* | 0.026 | 0.038 | 0.038 |
| *Sericornis nouhuysi* | 0.089 | 0.104 | 0.072 |

Table S6: Accession numbers of Genbank samples (not generated by us) used in *COI* gene network analysis.

| Taxon | GenBank accession number |
| --- | --- |
| *Aethomyias papuensis* | GU825788 |
| *Aethomyias perspicillatus* | GU825765 |
| *Sericornis nouhuysi* | GU825789 |
| *Neosericornis citreogularis* | GU825763 |
| *Sericornis magnirostris* | GU825764 |

Table S7: Priors used for coalescent modelling in DIYABC. See Fig. S4 for further details.

| Parameter | Prior space |
| --- | --- |
| Effective population size for constant model for *Aethomyias papuensis* and *Aethomyias perspicillatus* (Nc) | 1000–100,000 |
| Effective population size for constant model for *Sericornis nouhuysi* (Nc) | 1000–150,000 |
| Effective population size post expansion for *Aethomyias papuensis* and *Aethomyias perspicillatus* (N) | 1000–100,000 |
| Effective population size post expansion for *Sericornis nouhuysi* (N) | 1000–150,000 |
| Ancestral effective population size (NA) | 10–50,000 |
| Time of expansion under Model B (TEXP1) | 10–25,000 |
| Time of expansion under Model C (TEXP2) | 110,000–130,000 |

Table S8: Summary statistics used for DIYABC analyses.

| Summary statistic | Type |
| --- | --- |
| Proportion of monomorphic loci | Within a population |
| Mean gene diversity across polymorphic loci | Within a population |
| Variance of genetic diversity across polymorphic loci | Within a population |
| Mean gene diversity for all loci | Within a population |
